# Transposition of Tn*552-II* (a Tn*552* derivative) to the conjugative pCtra plasmid family in pediatric multidrug-resistant community-associated MRSA

**DOI:** 10.1128/aac.00647-25

**Published:** 2025-09-03

**Authors:** Tsai-Wen Wan, Masaki Shintani, Kenji K. Kojima, Naohiko Moriguchi, Wei-Chun Hung, Yu-Ting Wang, Lee-Jene Teng, Tatsuo Yamamoto

**Affiliations:** 1 Department of Epidemiology, Genomics, and Evolution, International Medical Education and Research Center, Niigata, Japan; 2 Department of Clinical Laboratory Sciences and Medical Biotechnology, National Taiwan University College of Medicine 38005, Taipei City, Taiwan; 3 Department of Microbiology and Immunology, College of Medicine, Kaohsiung Medical University 38023, Kaohsiung, Taiwan; 4 Department of Environment and Energy Systems, Graduate School of Science and Technology, Shizuoka University 197502, Hamamatsu, Japan; 5 Genetic Information Research Institute (GIRI) 128762, Cupertino, California, USA; 6 Department of Pediatrics, Kaizuka City Hospital 73755, Kaizuka, Japan; 7 Division of Research and Analysis, Food and Drug Administration, Ministry of Health and Welfare 63123, Taipei, Taiwan; Shionogi Inc., Florham Park, New Jersey, USA

**Keywords:** community-associated methicillin-resistant *Staphylococcus aureus*, Tn*552-II*, transposition, circular intermediate, conjugative plasmid

## Abstract

We report the transposition of a 9,045-bp transposon (Tn*552-II*) carrying the *tnpABC*/target-gene-like(*tgl*)/*blaZ-R1-I* array to a conjugative plasmid (pCtra) in pediatric multidrug-resistant community-associated methicillin-resistant *Staphylococcus aureus*. Tn*552-II* contained a 7-bp target site (*att*) on the left; an internal *i-att* on the right, apparently “brought-in” through a circular intermediate; and *i-att2* and *i-att3* in the *tgl* gene and its neighboring gene, respectively. This transposition enhanced ampicillin resistance and facilitated Tn*552-II* spread at a frequency of 10^−3^.

## INTRODUCTION

Bacterial antimicrobial resistance is a major threat to global public health (1). Methicillin-resistant *Staphylococcus aureus* (MRSA) is a common multidrug-resistant pathogen (1, 2). MRSA includes healthcare-associated MRSA (HA-MRSA) and community-associated MRSA (CA-MRSA) (2), each of which can be classified into several sequence types (ST)/staphylococcal cassette chromosome *mec* (SCC*mec*) types (2–4). HA-MRSA includes ST239/SCC*mec*III (5) and ST5/SCC*mec*II (also termed the New York/Japan clone, NY/J) (3). CA-MRSA has received particular attention since the late 1990s and includes ST8/SCC*mec*IVa (USA300) (2). SCC*mec* type IV has replaced SCC*mec* type II HA-MRSA in MRSA bloodstream infections in Japan (6). SCC*mec* possesses the *mecA* gene, encoding resistance to methicillin or oxacillin (penicillinase-stable penicillin), imipenem (carbapenem), and cefoxitin (cefem) (7), and resistance levels are higher for HA-MRSA than CA-MRSA (8). Resistance to ampicillin (penicillinase-labile penicillin) is attributable to the *blaZ* gene encoding penicillinase (7).

Tn*552*, a transposon carrying the *blaZ-R1-I* array, is integrated into the ICE*6013* genetic element of HA-MRSA ST239/SCC*mec*III (9), and is epidemiologically associated with its global dissemination (5, 10, 11). We reported *blaZ-R1-I* (not associated with ICE*6013*) as the Tn*552* transposon in CA-MRSA in 2012 (12), albeit with its possible transposase region exhibiting no homology to Tn*552*. This CA-MRSA type (CA-MRSA/J) exhibited the major genotype of ST8/SCC*mec*IVl/*spa*t1767/*agr*1/coagulaseIII according to its clonal pulsed-field gel electrophoresis patterns and spread to Japan and Hong Kong. The genome data on strain NN50 revealed a Tn*552*CA-MRSA/J structure (12), and they were deposited in the GenBank database under accession number BAEA01000000 (contig_1). PCR data for strains NN3, NN4, and SI1 were obtained in 2019 (13). In 2020, Tn*552*CA-MRSA/J was detected in plasmid pW51A in pediatric multidrug-resistant CA-MRSA/J strain T51, possessing the *tnpABC*/target-gene-like(*tgl*)/*blaZ-R1-I* array. The aim of this study was to clarify the molecular features of the chromosome-to-plasmid transposition of Tn*552*CA-MRSA/J (hereafter referred to as Tn*552-II*), a Tn*552* derivative.

The CA-MRSA/J strains (*n* = 57), which were laboratory stock strains from previous clinical episodes (12–14) and related strains, were stored at −80°C. All strains we tested *blaZ* (Tn*552-II*)-positive by PCR (12, 13), and the minimum inhibitory concentrations (MICs) of antimicrobial agents were determined (7). The genetic and clinical characteristics of major strains are summarized in Table S1(15). Complete MRSA genome sequences were analyzed, and PCR was performed as described previously (13, 16).

The structures of plasmids pW34A, pW51A, and pSAJ1 are shown in Fig. S1, S2, and S3, respectively. pW34A and pW51A are members of the pCtra plasmid family of CA-MRSA/J, which is characterized by i) *repA* with a “SNH repeat” motif (HSNHSNH), ii) *parM* for plasmid partitioning of low-copy-number plasmids (17), iii) pSK41-type *tra* operon (containing genes for conjugative transfer) (18), and iv) multiple copies of IS*257*. pCtra originated from multidrug-resistant plasmids of NY/J in the 1970s–1980s (e.g., pSAJ1 in strain O3 (15) and pSK41 (18) from gentamicin-resistant *S. aureus* outbreaks in the USA in the 1970s).

**Fig 1:**
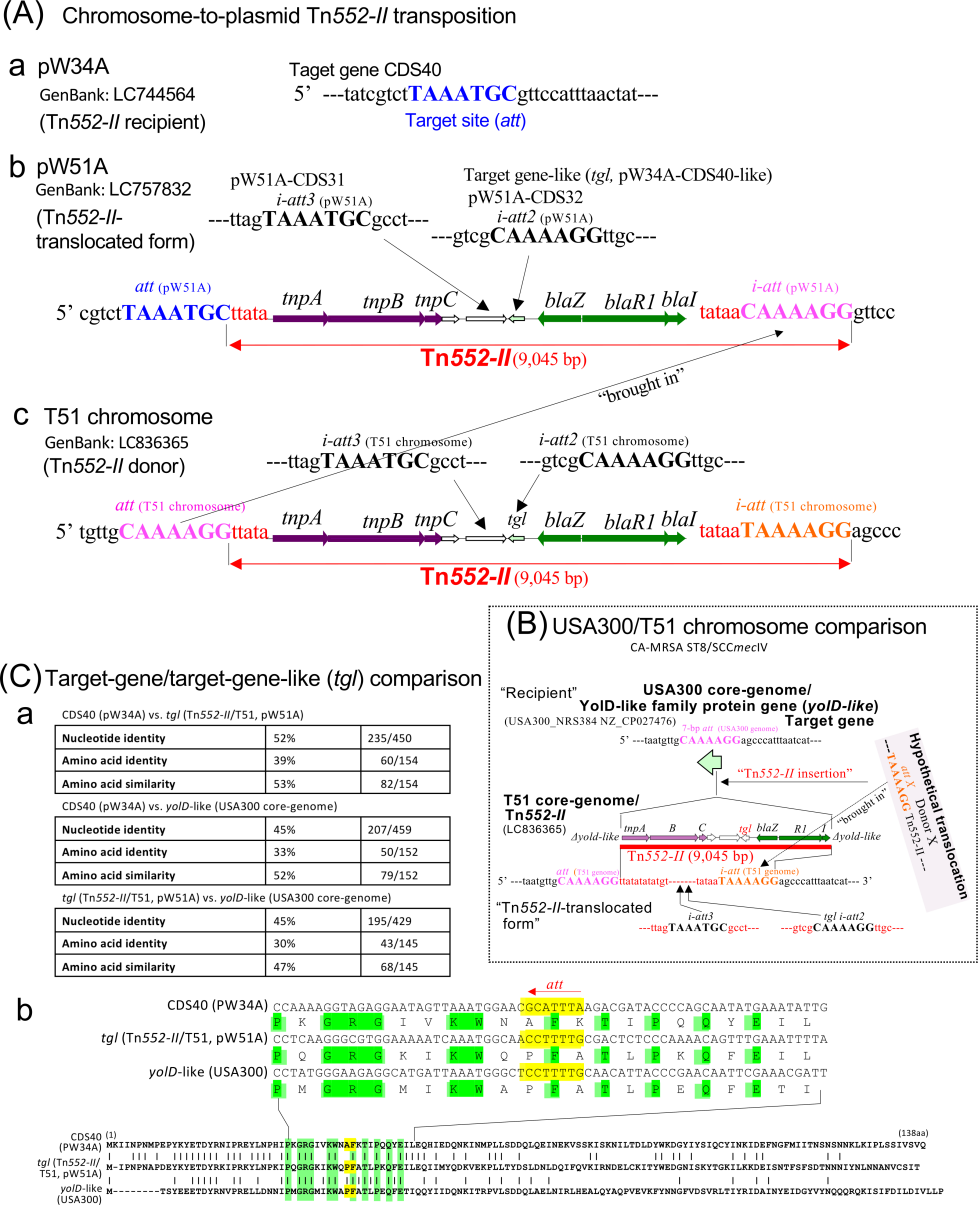
Complete genetic set related to a Tn*552-II* transposition event in CA-MRSA/J (**A**), summary of CA-MRSA chromosomal features related to Tn*552-II* (**B**), and comparison of the target gene (CDS40) of pW34A, *tgl* of Tn*552-II*, and the target gene of the USA300 chromosome (**C**). In (**A**), a 7-bp target site (*att*) sequence is shown in blue color. The inserted Tn*522-II* (shown in red color) contains the array *tnpABC*/*tgl*/blaZ-R1-I, (*tgl* stands for target-gene-like [CDS homologous to the target gene/CDS40pW34]). A different 7-bp target-site (*i-att*) sequence (shown in pink color) is present at the right-hand end of Tn*522-II*. The Tn*552-II* regions of plasmid pW51A and the T51 chromosome (as a Tn*552-II* donor) are the same, except for *att* and *i-att*, showing that the *att* (pink color) of the Tn*552-II*T51-chromosome is “brought-in” to the right-hand end of Tn*552-II*pW51A upon transposition. The Tn*552-II*T51-chromosome has a different 7-bp *i-att* (shown in orange color). Additional 7-bp *att* sequences in Tn*552-II* (*i-att2* present in *tgl* and *i-att3* present in the CDS next to *tgl*) are shown in black color. In (**B**), the chromosome of CA-MRSA ST8/SCC*mec*IVa (strain USA300) has a 7-bp *att* (pink color) in the YolD-like family protein gene (*yolD*-like). A depiction of the “*att*-brought-in” model, based on the USA300 “chromosomal *att*,” yielding the T51 chromosomal Tn*552-II*, is shown. In (**Ca**), identities of the genes at the nucleotide and amino acid sequence levels are summarized, and in the sequence alignments (**Cb**), the position of the 7-bp *att* site (yellow mark with a red arrow) is shown; the conserved amino acid sequences in each gene product around the 7-bp *att* position are shown in green color. Regarding *tnpABC* of Tn*552-II*, possible endonuclease motifs are: TnpA, HH/HPH/HTH; TnpB, H--H/H---H; TnpC, SKH.

Plasmid pW51A showed some genetic changes compared with pW34A (Fig. S4). Notably, pW51A had a Tn*552-II* insertion at a coding sequence (CDS) (target gene), which corresponded to CDS40pW34A (Fig. S4,1Aa and b). CDS40pW34A was identical to CDS50pSAJ1 (Fig. S3), indicating that the target gene originated in NY/J. The target gene had a 7-bp target-site (*att*) sequence (TAAATGC); Tn*552-II* was inserted at the 3ʹ end of *att* (Fig. S4B,1Ab). Tn*552-II* was 9,045 bp in size and had a different *att* (CAAAAGG) at its right-hand end, an internal *att* (*i-att*) (Fig. 1Ab). *tgl* showed homology to CDS40pW34A and had an *att* copy (CAAAAGG) as the second *i-att* (*i-att2*), corresponding to a “(CAAAAGG)-*blaZ-R1-I*-(CAAAAGG)” structure (Fig. 1Ab). An additional *i-att* motif (TAAATGC), designated *i-att3*, was identified within CDS31pW51A, corresponding to the *att* site of Tn*552-II* (Fig. 1Ab).

Figure S5, 1Ab and c show a comparison of the Tn*552-II* region in pW51A and the T51 chromosome. The two regions were identical, except for alterations in *att* and *i-att*. pW51A had therefore acquired Tn*552-II* from the T51 chromosome, with the donor *att* sequence (CAAAAGG) being “brought-in” to the right end as *i-att* (CAAAGG). This “*att*-brought-in” model is consistent with Tn*554* (10, 19). No changes occurred to *tgl*, including *i-att2* and *i-att3*, which may be remnants of an ancestral form of Tn*552-II* that lacked the *bla* genes. A similar event may have occurred at the *yold-like* (*att*, CAAAAGG) site of CA-MRSA ST8/SCC*mec*IV, such as in USA300, to yield a CA-MRSA/J (T51) chromosome carrying Tn*552-II* (Fig. 1B), albeit with possible changes in *i-att2* and *i-att3*.

High levels of homology were observed between CDS40pW34A and the *tgl* gene, particularly in the N-terminal region, but *yold-like-*USA300 (the chromosomal target gene) showed divergence from these two regions (Fig. 1Ca and b). The *att* or *i-att2* sequences were located at similar positions, with some conserved amino acid sequences (Fig. 1Cb), indicating that CDS40pW34A and *tgl* may play a combined role in the Tn*552-II* transposition event. Moreover, *tgl* may provide a rescue mechanism for the function of target genes disrupted by Tn*552-II* insertion.

**Fig 2:**
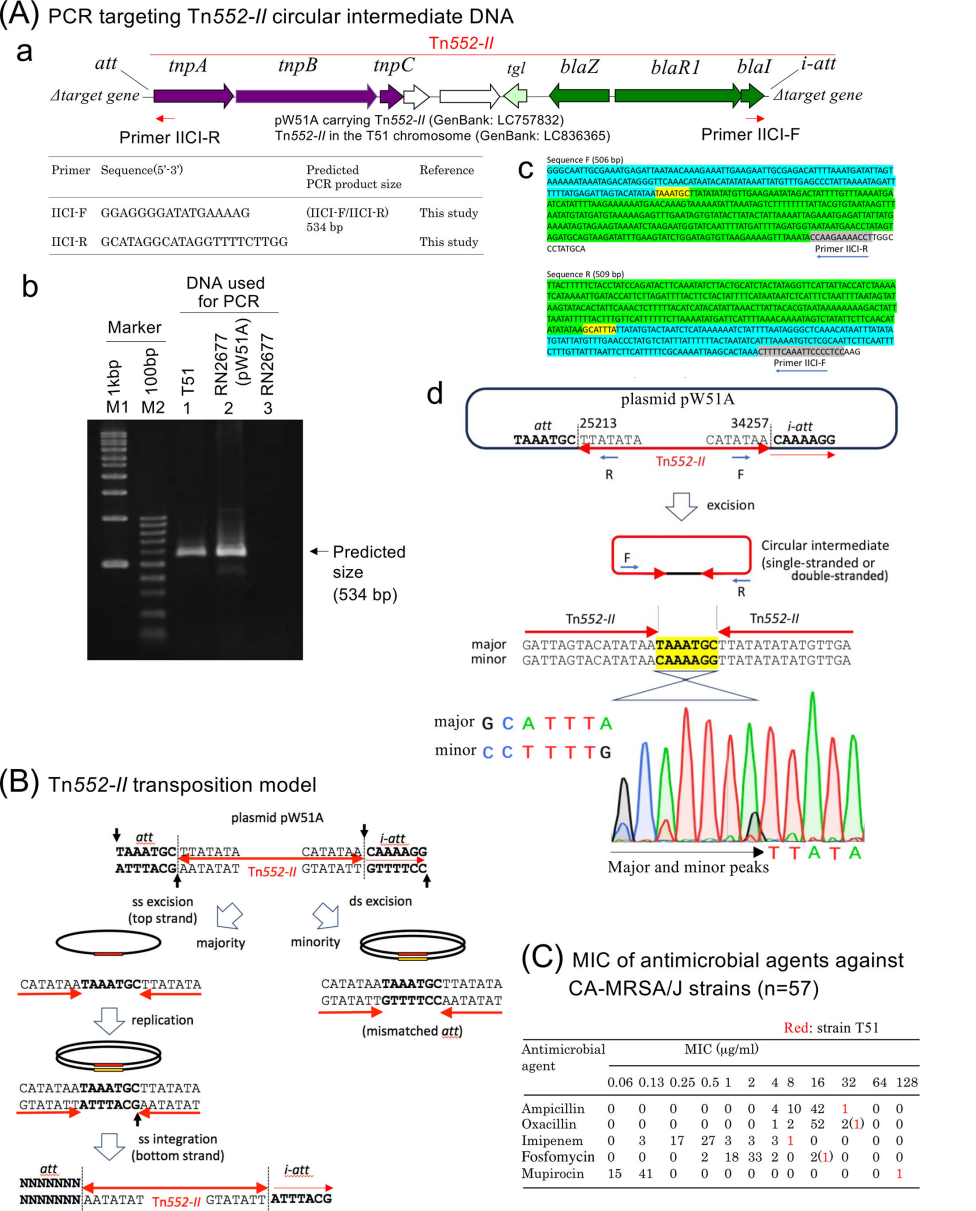
Detection and characterization of the transposition circular intermediate of Tn*552-II* (**A**), a model of transposition through a circular intermediate for Tn*552-II* (**B**), and the MICs of five antimicrobial agents against 57 CA-MRSA/J strains (**C**). In (**A**), a PCR primer set (IICI-F and IICI-R) to detect a circular intermediate was designed in (a), which successfully yielded PCR products (circular intermediate of Tn*552-II*) with an expected size (b) and expected sequences (c) for both the donor strain T51 and transconjugant RN2677 carrying pW51A; in (c), for the sequences determined from the IICI-F and IICI-R primers, the *att* sequence is shown in yellow, the right terminal sequence of Tn*552-II* is colored blue, and the left side is colored green (each sequence reached the opposite primer position, shown in gray), and in (d), regarding a 7-bp spacer between the termini of Tn*552-II*, two sequences were detected, the major sequence TAAATGC, corresponding to *att* (at the left junction) and the minor sequence CAAAAGG, corresponding to *i-att* (at the right junction), suggesting that there are two types of circular intermediates, revealing the biased incorporation of *att* over *i-att* in the circular transposition intermediate. In (**B**), based on the experimental data (c and d), a model of Tn*552-II* transposition is presented. In (**C**), MIC data of strain T51 are indicated in red, representing a multidrug-resistant strain isolated from a pediatric patient.

The circular transposition intermediate of Tn*552-II* was validated by PCR and sequencing (Fig. 2A), as previously described for Tn*554* (10, 19). Between the termini of Tn*552-II*, there was a 7-bp spacer. The spacer sequence was predominantly TAAATGC, corresponding to *att* of the donor plasmid, but a low frequency of the sequence CAAAAGG was also detected, corresponding to *i-att* (Fig. 2Ad). This supports the biased incorporation of *att* over *i-att* in the circular transposition intermediate; the biased incorporation of the 5ʹ flanking sequence in circular intermediates was reported for “strand-biased circularizing integrative elements” (SEs) (20, 21).

A transposition model of Tn*552-II* is shown in Fig. 2B. The top strand exchange site is assumed at the 5ʹ end of *att/i-att*, and the bottom strand exchange site is at the 3ʹ end of *att/i-att*. For the recombination mediated by the tyrosine recombinase tetramer, the “half-of-the-sites” activity accounts for the two-step, single-strand exchange mechanism of recombination (20, 21). Therefore, the top strand exchange precedes the bottom strand exchange, resulting in single-stranded excision of the top strand only (containing *att*). At low frequency, double-stranded excision would occur, resulting in a mismatched spacer derived from both *att* and *i-att*. The single-stranded circular intermediate would then be replicated, with the resulting double strand containing a spacer harboring *att*. To accomplish “*att*-brought-in,” only the bottom strand would be integrated into the target DNA sequence, leading to single-stranded transposition of Tn*552-II*. Each step of transposition remains to be validated experimentally.

Plasmid transfer was performed as described previously (10, 11). pW51A, which confers resistance to mupirocin and ampicillin, was transferred at high frequency (1.5 × 10^−3^) in filter mating, and low frequency (< 1.0 × 10^−8^) without membrane filters, indicating that pW51A efficiently disseminates Tn*552-II*. In contrast, the transfer frequency of Tn*554* (*ermA*) in filter mating was 10^−7^ (Table S1).

Tn*552-II* differs from Tn*552* (Fig. S6A) and Tn*554* (Fig. S6B). PCR targeting Tn*552-II* (*tnpABC* and *blaZ-R1-I*) detected all CA-MRSA/J strains (Fig. S6Ca, lanes 1–8; Fig. S6Cb, lane 1); only *blaZ-R1-I* of Tn*552* (Fig. S6C-a, lanes 11 and 12; Fig. S6Cb, lanes 2 and 3); and did not detect Tn*554* (Fig. S6Ca, lanes 13–15).

Among CA-MRSA/J strains (*n* = 57), strain T51 exhibited high MIC values for ampicillin, oxacillin, imipenem, fosfomycin, and mupirocin (Fig. 2C). These high MICs may be caused by: for ampicillin, two copies of Tn*552-II* carrying *blaZ* (on the chromosome and pW51A); for mupirocin, *mupA* (in pW51A); and for fosfomycin, *fosB* (on the chromosome). High MICs for imipenem (intermediate) and oxacillin (reaching HA-MRSA levels) (8) may result from changes in penicillin-binding protein (7). β-Lactam agents, fosfomycin, gentamicin, and mupirocin have been widely used in pediatric treatment and for MRSA eradication in Japan. This highlights the significance of plasmid dissemination and Tn*552-II* in the spread of drug resistance.

Regarding Tn*552-II*, similar data were published (22) during the course of the present study; however, our data are based on precise sequences before and after transposition and focus on the chromosome-to-plasmid transposition through a circular intermediate.

## Data Availability

The DNA sequences have been deposited in the GenBank database under the accession numbers LC744564, LC757832, LC755502, LC836365, LC843447, and LC843446.

## SUPPLEMENTAL MATERIAL

Supplemental figures aac.00647-25-s0001.docx Fig. S1 to S6.

Table S1 aac.00647-25-s0002.docx Relevant characteristics of CA-MRSA ST8/SCCmecIVl (CA-MRSA/J) strains, HA-MRSA strains, and *S. aureus* strains employed in this study.

ASM does not own the copyrights to Supplemental Material that may be linked to, or accessed through, an article. The authors have granted ASM a non-exclusive, world-wide license to publish the Supplemental Material files. Please contact the corresponding author directly for reuse.

## References

[1] World Health Organization. 2023. Antimicrobial resistance. Geneva World Health Organization. https://www.who.int/news-room/fact-sheets/detail/antimicrobial-resistance.

[2] Otto M. 2013. Community-associated MRSA: what makes them special? Int J Med Microbiol 303:324–330. doi:10.1016/j.ijmm.2013.02.007 23517691 PMC3729626

[3] Yamamoto T, Hung WC, Takano T, Nishiyama A. 2013. Genetic nature and virulence of community-associated methicillin-resistant Staphylococcus aureus. Biomedicine (Taipei) 3:2–18. doi:10.1016/j.biomed.2012.12.001

[4] International Working Group on the Classification of Staphylococcal Cassette Chromosome Elements (IWG-SCC). 2009. Classification of staphylococcal cassette chromosome mec (SCCmec): guidelines for reporting novel SCCmec elements. Antimicrob Agents Chemother 53:4961–4967. doi:10.1128/AAC.00579-09 19721075 PMC2786320

[5] Harris SR, Feil EJ, Holden MTG, Quail MA, Nickerson EK, Chantratita N, Gardete S, Tavares A, Day N, Lindsay JA, Edgeworth JD, de Lencastre H, Parkhill J, Peacock SJ, Bentley SD. 2010. Evolution of MRSA during hospital transmission and intercontinental spread. Science 327:469–474. doi:10.1126/science.1182395 20093474 PMC2821690

[6] Kaku N, Sasaki D, Ota K, Miyazaki T, Yanagihara K. 2022. Changing molecular epidemiology and characteristics of MRSA isolated from bloodstream infections: nationwide surveillance in Japan in 2019. J Antimicrob Chemother 77:2130–2141. doi:10.1093/jac/dkac154 35639590

[7] Clinical and Laboratory Standards Institute. 2021. Performance standard for antimicrobial susceptibility testing. Vol. Supplement M100-Ed31. Wayne PA Clinical and Laboratory Standards Institute

[8] Takano T, Higuchi W, Yamamoto T. 2009. Superior in vitro activity of carbapenems over anti-methicillin-resistant Staphylococcus aureus (MRSA) and some related antimicrobial agents for community-acquired MRSA but not for hospital-acquired MRSA. J Infect Chemother 15:54–57. doi:10.1007/s10156-008-0665-5 19280303

[9] Smyth DS, Robinson DA. 2009. Integrative and sequence characteristics of a novel genetic element, ICE6013, in Staphylococcus aureus. J Bacteriol 191:5964–5975. doi:10.1128/JB.00352-09 19648240 PMC2747909

[10] Yamamoto T, Takano T, Higuchi W, Iwao Y, Singur O, Reva I, Otsuka Y, Nakayashiki T, Mori H, Reva G, Kuznetsov V, Potapov V. 2012. Comparative genomics and drug resistance of a geographic variant of ST239 methicillin-resistant Staphylococcus aureus emerged in Russia. PLoS ONE 7:e29187. doi:10.1371/journal.pone.0029187 22276107 PMC3261861

[11] Khokhlova OE, Hung WC, Wan TW, Iwao Y, Takano T, Higuchi W, Yachenko SV, Teplyakova OV, Kamshilova VV, Kotlovsky YV, Nishiyama A, Reva IV, Sidorenko SV, Peryanova OV, Reva GV, Teng LJ, Salmina AB, Yamamoto T. 2015. Healthcare- and community-associated methicillin-resistant Staphylococcus aureus (MRSA) and fatal pneumonia with pediatric deaths in krasnoyarsk, Siberian Russia: unique MRSA’s multiple virulence factors, genome, and stepwise evolution. PLoS ONE 10:e0128017. doi:10.1371/journal.pone.0128017 26047024 PMC4457420

[12] Iwao Y, Ishii R, Tomita Y, Shibuya Y, Takano T, Hung WC, Higuchi W, Isobe H, Nishiyama A, Yano M, Matsumoto T, Ogata K, Okubo T, Khokhlova O, Ho PL, Yamamoto T. 2012. The emerging ST8 methicillin-resistant Staphylococcus aureus clone in the community in Japan: associated infections, genetic diversity, and comparative genomics. J Infect Chemother 18:228–240. doi:10.1007/s10156-012-0379-6 22350401

[13] Wan TW, Teng LJ, Yamamoto T. 2019. Structures of a highly variable cell-wall anchored protein-encoding the spj gene from ST8/SCCmecIVl community-associated methicillin-resistant Staphylococcus aureus (CA-MRSA/J) isolated from 2003 onwards: An indicator of a strongly invasive pathotype. Microbiol Immunol 63:186–193. doi:10.1111/1348-0421.12684 31009089 PMC6617794

[14] Ishitobi N, Wan TW, Khokhlova OE, Teng LJ, Yamamori Y, Yamamoto T. 2018. Fatal case of ST8/SCCmecIVl community-associated methicillin-resistant Staphylococcus aureus infection in Japan. New Microbes New Infect 26:30–36. doi:10.1016/j.nmni.2018.08.004 30245831 PMC6141726

[15] Yamamoto T, Tamura Y, Yokota T. 1988. Antiseptic and antibiotic resistance plasmid in Staphylococcus aureus that possesses ability to confer chlorhexidine and acrinol resistance. Antimicrob Agents Chemother 32:932–935. doi:10.1128/AAC.32.6.932 3415214 PMC172311

[16] Wan TW, Khokhlova OE, Iwao Y, Higuchi W, Hung WC, Reva IV, Singur OA, Gostev VV, Sidorenko SV, Peryanova OV, Salmina AB, Reva GV, Teng LJ, Yamamoto T. 2016. Complete circular genome sequence of successful ST8/SCCmecIV community-associated methicillin-resistant Staphylococcus aureus (OC8) in Russia: one-megabase genomic inversion, IS256’s spread, and evolution of Russia ST8-IV. PLoS ONE 11:e0164168. doi:10.1371/journal.pone.0164168 27741255 PMC5065196

[17] Popp D, Xu W, Narita A, Brzoska AJ, Skurray RA, Firth N, Goshdastider U, Maéda Y, Robinson RC, Schumacher MA. 2010. Structure and filament dynamics of the pSK41 actin-like ParM protein: implications for plasmid DNA segregation. J Biol Chem 285:10130–10140. doi:10.1074/jbc.M109.071613 20106979 PMC2843175

[18] Berg T, Firth N, Apisiridej S, Hettiaratchi A, Leelaporn A, Skurray RA. 1998. Complete nucleotide sequence of pSK41: evolution of staphylococcal conjugative multiresistance plasmids. J Bacteriol 180:4350–4359. doi:10.1128/JB.180.17.4350-4359.1998 9721269 PMC107441

[19] . Churchward G. 2002. Conjugative transposons and related mobile elements, p 177-191. In Craig NL, Craigie R, Gellert M, Lambowitz AM (ed), Mobile DNA II. ASM Press, American Society for Microbiology, Washington, DC

[20] Nonaka L, Masuda M, Yano H. 2022. Atypical integrative element with strand-biased circularization activity assists interspecies antimicrobial resistance gene transfer from Vibrio alfacsensis. PLoS ONE 17:e0271627. doi:10.1371/journal.pone.0271627 35917316 PMC9345347

[21] Idola D, Mori H, Nagata Y, Nonaka L, Yano H. 2023. Host range of strand-biased circularizing integrative elements: a new class of mobile DNA elements nesting in Gammaproteobacteria. Mob DNA 14:7. doi:10.1186/s13100-023-00295-5 37237359 PMC10214605

[22] Krüger H, Ji X, Wang Y, Feßler AT, Wang Y, Wu C, Schwarz S. 2021. Identification of Tn553, a novel Tn554-related transposon that carries a complete blaZ-blaR1-blaI β-lactamase operon in Staphylococcus aureus. J Antimicrob Chemother 76:2733–2735. doi:10.1093/jac/dkab210 34164661

