# Supplementary material for: Transposition of Tn552-II (a Tn552 derivative) to the conjugative pCtra plasmid family in pediatric multidrug-resistant community-associated MRSA
Source: Antimicrob Agents Chemother. 2025 Sep 3;69(10):e00647-25. doi: 10.1128/aac.00647-25 (PMC12486829; doi:10.1128/aac.00647-25)
Supplement: Supplemental figures — Fig. S1 to S6. [file aac.00647-25-s0001.docx]

FIG S1 Structure of pW34A. Characteristics of pW34A: *repA* with a motif of SNH repeats (HSNHSNH); *tra1-tra13* (type IV secretion system-mediated transfer genes) (shwon in blue), *oriT* (origin transfer), and *mobQ* (MOB_Q_ type of relaxase/*oriT* nickase) for conjugative transfer; *parM* for plasmid partition (with actin-like filament/ATPase); multiple copies (#1-#6) of IS*257* (shown in purple); and *aacA-aphD* in Tn*4001* (with ΔIS*256*), *bleO*, and *aadD* for drug resistance. Coding sequence 40 (CDS40) is the target gene of Tn*552-II* transposition. CDS40 product: N-acetylglucosamine-1-phosphateuridyltransferase. containing nucleotidyltransferase and isoleucine patch surperfamily acetyltransferase domains, or YolD-like family protein. GenBank accession number: LC744564.

FIG S2 Structure of pW51A. Characteristics of pW51A: *repA*, *tra1-tra13*, *oriT*, *mobQ*, and *parM*, similar to pW34A (FIG S1); four copies of IS*257*; and *aadD, mupA, and blaZ-R1-I in Tn552-II* for drug resistance. Tn*552-II* is inserted into the target gene (corresponding to CDS40 of pW34A), yielding split genes (CDS26 and CDS36). pW51A lacks Tn*4001* (with ΔIS*256*) carrying *aacA-aphD*. GenBank accession number: LC757832.

FIG S3 Structure of pSAJ1. GenBank accession number: LC755502. pSAJ1 is a plasmid of HA-MRSA New York/Japan (NY/J) clone (strain O3), isolated in 1986. A region indicated by an outer red line, named pW34A, is shared by pSAJ1 and pW34A; thus, pSAJ1 has *repA*, *tra1-tra13*, *oriT*, *mobQ*, *parM, bleO, aacA-aphD* in Tn*4001* (with ΔIS*256*), and the target gene for Tn*552-II* transposition (CDS50pSAJ1), similar to pW34A*.* A region indicated by an outer black thick line, named VRSAp, is shared by pSAJ1 and a plasmid (VRSAp) of vancomycin-resistant HA-MRSA New York/Japan clone (strain Mu50) (GenBank accession number: AP003367), covering *mobQ*, *qacA*, Tn*4001* (with ΔIS*256*) carrying *aacA-aphD*, and Tn*552-II-*target gene. Thus, the target gene for Tn*552-II* originates in a NY/J plasmid.

FIG S4 Comparison of pW34A and pW51, revealing the structure of Tn*552-II.* Plasmids pW34A and pW51A are members of the pCtra plasmid family of CA-MRSA/J, pW34A and pW51A being “Tn*552-II* recipient” and “Tn*552-II*-harboring plasmid”, respectively. In (A), changes in pW51A (compared with pW34A) are summarized in a light pink zone (above the pW34A structure). More precise comparison is shown in (B). Coding sequence 40 (CDS40) of pW34A (CDS40pW34A) is a target gene for Tn*552-II* transposition and includes a 7-bp target site (*att*) sequence (shown in blue). Tn*552-II* is shown in red (and the *tnp* and *bla* genes are shown in purple and green, respectively). The insertion of Tn*552-II* into the target gene in pW51A results in two split CDSs, CDS26 and CDS36 (B). The inserted Tn*522-II* has the array *tnpABC*/*tgl*/blaZ-R1-I, (*tgl* stands for target-gene-like [CDS homologous to the target gene/CDS40pW34]). A different 7-bp target-site (*i*-*att*) sequence (shown in pink) is present at the right-hand end of Tn*522-II*. *tgl* (CDS32pW51A) also has a 7-bp *att* (*i*-*att2*); and its neighboring CDS (CDS31pW51A) has a 7-bp *att* (*i*-*att3*).

FIG S5 Tn*552-II* regions of pW51A and the T51 chromosome. Tn*552-II* regions are shown in red; and the *tnp* and *bla* genes are shown in purple and green, respectively. The two Tn*552-II* regions are the same, except for *att* and *i*-*att*, showing that the *att* of the Tn*552-II* T51-chromosome (shown in pink) is “brought in” to the right-hand end of Tn*552-II*pW51A (shown in pink) upon transposition. The *att* of Tn*552-II* pW51A (shown in blue) corresponds to that of the target gene (CDS40) of pW34A (Tn*552-II*-recipient). The *tgl* copies (with *i*-*att2*) of Tn*552-II*T51-chromosome and Tn*552-II*pW51A, shown in black, are the same; the CDS copies (with *i*-*att3*), which are located next to *tgl*, of Tn*552-II*T51-chromosome and Tn*552-II*pW51A, shown in black, are also the same.

FIG S6 The structures of Tn*552* (present in ICE*6013* in the HA-MRSA ST239/SCC*mec*III chromosome, A) and Tn*554* (present in the CA-MRSA/J SI1 chromosome, B), and PCR targeting the *tnp* and *bla* of Tn*552-II* (C). In (A) and (B), transposition- and drug resistance-related genes are shown in purple and green, respectively. In (A), *p480* (*tnpA*) was splitted in strain 16K (*p480-1* [*tnpA1*] and *p480-2* [*tnpA2*])*.* In (B), the chromosome of CA-MRSA/J (for example, strain NN3) had 6-bp *att* (shwon in blue) in *radC*, and in strain SI1, Tn*554* was inserted at this *att* site; however, in the SI1 case, inserted Tn*554* had the same 6-bp sequence (GATGTA) at *att* and *i-att.* In (C), Tn*552-II’s* *tnp* and *bla* were tested for CA-MRSA/J strains (carrying Tn*552-II*), HA-MRSA ST239/SCC*mec*III strains (carrying Tn552), and *S*. *aureus* RN2677 carrying Tn*554,* which was obtained from HA-MRSA ST239/SCC*mec*III strains (16K, OC3) and a CA-MRSA/J strain (SI1).
