# Supplementary material for: Transposition of Tn552-II (a Tn552 derivative) to the conjugative pCtra plasmid family in pediatric multidrug-resistant community-associated MRSA
Source: Antimicrob Agents Chemother. 2025 Sep 3;69(10):e00647-25. doi: 10.1128/aac.00647-25 (PMC12486829; doi:10.1128/aac.00647-25)
Supplement: Table S1 — Relevant characteristics of CA-MRSA ST8/SCCmecIVl (CA-MRSA/J) strains, HA-MRSA strains, and S. aureus strains employed in this study. [file aac.00647-25-s0002.docx]

Table S1 Relevant characteristics of CA-MRSA ST8/SCC*mec*IVl (CA-MRSA/J) strains, HA-MRSA strains, and *S*. *aureus* strains employed in this study.

| Strain  (alternative  name) | MRSA genotype  ST/SCC*mec*/  *spa*/*agr*/*coa* | pCtra family  plasmid  (size in bp) | Transposon | | | Genome sequence analysis:  year | Patient age | Disease manifestations (admission or outpatient)  or information on strain construction in vitro | Reference |
| --- | --- | --- | --- | --- | --- | --- | --- | --- | --- |
|  |  |  | Tn*552-II*  (location) | Tn*554*  (location) | Tn*552*  (location) |  |  |  |  |
| **CA-MRSA/J** | | |  | | |  | |  |  |
| T34 (809) | 8/IVl/  t1767/1/III | +  pW34A  (42,576) | + (chromosome) | - | - | 2020 | 5M | Carrier/urine (outpatient) | 12, 13, this study |
| T51 (43) | 8/IVl/  t1767/1/III | +  pW51A  (45,096) | +  (chromosome and pW51A) | - | - | 2020/2023 | 2M | Pharynx colonization/carrier (admission for pneumonia) | This study |
| NN50 | 8/IVl/  t1767/1/III | - | + (chromosome) | - | - | 2011/2020 | 11Y | Abscesses at erector spinae muscles and epidural region of spine (an influenza prodrome case, a student taking physical education classes) (admission) | 12, 13, this study |
| NN55 | 8/IVl/  t1767/1/III | - | + (chromosome) | - | - | 2020 | 62Y | Iliopsoas abscesses and discitis with systemic myalgia and thrombocytopenia | 13, this study |
| SI1 | 8/IVl/  t1767/1/III | - | + (chromosome) | + (chromosome) | - | 2016/2020 | 64Y | Iliopsoas abscesses, MRSA sepsis, disseminated intravascular coagulation, multiorgan failure, septic pulmonary embolism, toxic shock syndrome (a vancomycin-non-responding case) (admission) | 13, 14, this study |
| T4 (KS3) | 8/IVl/  t1767/1/III | - | + (chromosome) | - | - | 2020/2023 | 8Y | MRSA sepsis, N/A, not available (admission) | This study |
| NN3 | 8/IVl/  t12625/1/III | - | + (chromosome) | - | - | 2023 | 1Y | Bullous impetigo (outpatient) | 12, 13, this study |
| NN4 | 8/IVl/  t1767/1/III | - | + (chromosome) | - | - | 2020 | 7M | Bullous impetigo (outpatient) | 12, 13, this study |
| **HA-MRSA** | |  |  |  |  |  |  |  |  |
| O3 | 5/II(2A)/  t002/2/II | +  pSAJ1  (52,653) | - | - | - | 2021 | N/A, not available | Chronic respiratory failure (admission) | 15, this study |
| 16K | 239/III.1.1.4R/  351(t030)/1/IV | - | - | + (chromosome) | + (chromosome) | 2010 | 19-40 Y | Urethritis (outpatient) | 10, this study |
| OC3 | 239/III.1.1.2/  3(t037)/1/IV | - | - | + (chromosome) | + (chromosome) | 2014 | 46 Y | Pneumonia, MRSA sepsis | 11, this study |
| **Other *S. aureus*** | |  |  |  |  |  |  |  |  |
| RN2677 carrying Tn*554* | - | - | - | + (chromosome) | - | - | - | RN2677 carrying Tn*554* (*ermA*) was constructed by bacterial filter mating between Tn*554*-positive MRSA (SI1, 16K, or OC3) and RN2677; transfer frequency, 10^-7^ | 10, 11, 13, this study |
